# Protein control of membrane and organelle dynamics: Insights from the divergent eukaryote *Toxoplasma gondii*

**DOI:** 10.1016/j.ceb.2022.102085

**Published:** 2022-06

**Authors:** Jana Ovciarikova, Rodolpho Ornitz Oliveira Souza, Gustavo Arrizabalaga, Lilach Sheiner

**Affiliations:** 1 Wellcome Centre for Integrative Parasitology, University of Glasgow, Glasgow G12 8TA, UK; 2 Department of Pharmacology and Toxicology, Indiana University School of Medicine, Indianapolis, IN, USA

## Abstract

Integral membrane protein complexes control key cellular functions in eukaryotes by defining membrane-bound spaces within organelles and mediating inter-organelles contacts. Despite the critical role of membrane complexes in cell biology, most of our knowledge is from a handful of model systems, primarily yeast and mammals, while a full functional and evolutionary understanding remains incomplete without the perspective from a broad range of divergent organisms.

Apicomplexan parasites are single-cell eukaryotes whose survival depends on organelle compartmentalisation and communication. Studies of a model apicomplexan, *Toxoplasma gondii,* reveal unexpected divergence in the composition and function of complexes previously considered broadly conserved, such as the mitochondrial ATP synthase and the tethers mediating ER–mitochondria membrane contact sites. Thus, *Toxoplasma* joins the repertoire of divergent model eukaryotes whose research completes our understanding of fundamental cell biology.

## Introduction

Membrane bound complexes play key roles in the control of organelle function through mediating interactions and exchange between membranes and through defining membrane shape. Some complexes mediate interactions between membranes of the same organelle, intraorganellar contacts, such as the mitochondrial cristae, which creates microenvironments needed for functional control of organellar pathways. Other complexes mediate interactions between membranes of different organelles, enabling organellar cooperation in biosynthetic pathways and in controlling cellular ion and metabolite levels, such as the ER–mitochondrion membrane contact sites (MCS)s. While these roles are fundamental for eukaryotic cell biology, most of our current knowledge is focused on commonly studied organisms that are often closely related to each other in the perspective of the full tree of eukaryotes (Box 1).

Box 1 **The importance and benefits of understanding cell biology in divergent Organisms.**

The scheme herein is drawn after [1] and shows eukaryotic clades (blue) and model organisms (black and icon) mentioned in this review. The study of divergent organisms is an important goal for a cell biology community that strives to have knowledge of the full repertoire of functions and mechanisms taking place in eukaryotes.

**Figure:**
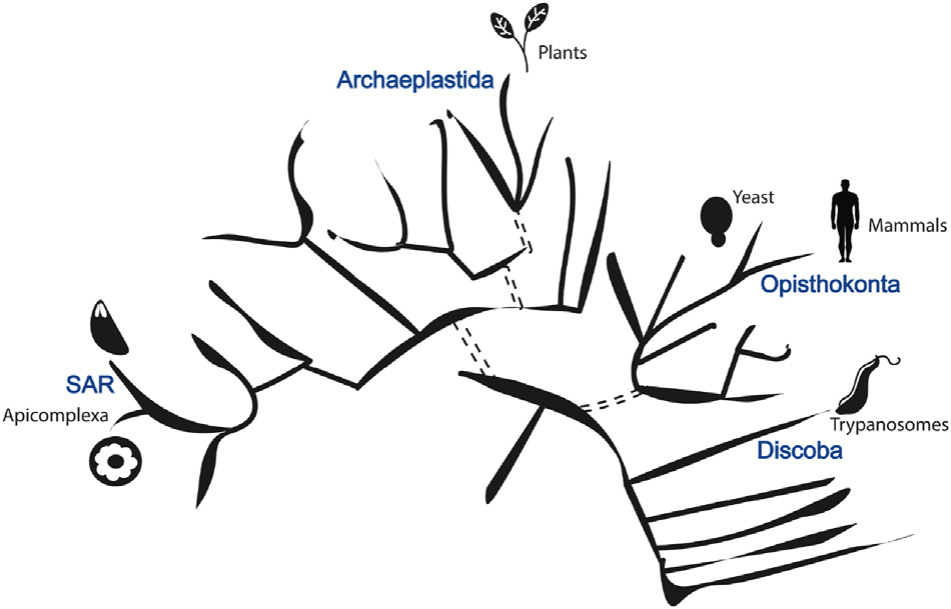

In addition to this critical basic scientific outcome, comparing the cell biology of organisms from different clades is a powerful tool to identify core features of cellular pathways common to all eukaryotes, to discover new functions of known machineries of well-studied organisms, and to obtain understanding of common functional mechanisms.

For example, distant organisms may employ proteins that are unrelated at the sequence level to perform the same cellular function. Comparing the structural feature of such proteins highlights the structural elements required for function, as was shown with the role of the mitochondrial outer membrane importer Mim1/2 from yeast and its functional homolog from trypanosomes pATOM3 [3].

In another example, the study of divergent organisms can demonstrate that a machinery found in yeast but not metazoan, was lost in metazoan rather than being yeast specific. The ER–mitochondria encounter structure (ERMES), was initially considered fungi specific, but then found in *Trypanosoma* and amoeba [4,5]. ERMES's role in trypanosomes is likely different from yeast [6], and amoeba have highly divergent mitochondria-like organelle, suggesting that there are still functions of ERMES that are unknown.

Alt-text: Box 1

For example, yeast and mammals are the subjects of numerous studies; however, both belong to the Opisthokont clade. Here we describe recent progress in our understanding of the role of membrane complexes in mediating membrane interactions, shape, and function, in the divergent eukaryote, *Toxoplasma gondii*, a model organism for the apicomplexan phylum, of the SAR clade (Box 1).

## Intraorganellar contacts: Complexes mediating mitochondrial cristae biogenesis and shape

One of the hallmarks of mitochondria is their double membrane composed of an outer and inner membrane. The latter folds to form cristae, whose shape and dimension control mitochondrial respiration [7], and which provide spatial organisation for the assembly of the mitochondrial electron transport chain complexes into super complexes [8]. Cristae morphology is mediated by three key protein complexes (reviewed here [9] in more details):

(I) Assemblies of the mitochondrial genome maintenance protein (Mgm1 or OPA1 in yeast or metazoans, respectively) stabilise and tighten the cristae neck. No homologs of Mgm1/OPA1 can be found in the *Toxoplasma* genome [10] and whether this role has been replaced by parasite-specific components remains to be discovered.

(II) The F_o_F_1_-ATP synthase typically forms rows of dimers that induce membrane curvature to establish cristae [11]. Interestingly, *Toxoplasma* mitochondria have a bulbous cristae shape, divergent from the predominantly lamellar cristae found in opisthokonts. Moreover, *Toxoplasma* ATP synthase contains numerous novel subunits only found in apicomplexans and other members of the SAR clade [12]). A recent structural study provided answers to both these puzzles. Tomography studies found that the *Toxoplasma* ATP synthase assembles into hexamers, which are the basis for higher order pentagonal pyramids that dictate the unique cristae shape [13]). Single particle analysis via CryoEM further found that this unique hexamerisation is mediated through interactions between some of the new parasite subunits in the lumenal region of the complex [13]. These findings provided an exciting example of how divergent organisms evolve novel solutions to fundamental cell biology needs.

The ATP synthase is not the only *Toxoplasma* mitochondrial complex that has many new subunits not found in opisthokonts. All three others respiratory complexes found in apicomplexans (II, III, and IV) have divergent compositions, and each complex consists of new subunits not seen in organisms from other clades [14]. In opisthokonts, complexes of the electron transport chain assemble into supercomplexes, which enhances the efficiency of electron transfer and the stability of the individual complexes [15]. Evidence for supercomplex formation is emerging for *Toxoplasma* [16] and the related apicomplexan, *Plasmodium falciparum* [17]. It is possible that some of the new subunits are involved in supercomplex formation, much like the role of new ATP synthase subunits in mediating hexamerisation; however, this remains to be experimentally addressed. Revealing the functions of the unique respiratory complex subunits is an outstanding and important task for the field, as a novel function not seen in other eukaryotes may unravel.

(III) The mitochondrial contact site and cristae organisation system (MICOS) is a multi-subunit assembly responsible for cristae junction formation and for contacts with the outer mitochondrial membrane. MICOS is found across the eukaryotic tree with two core subunits found in all studied clades: MIC60 and MIC10 [18]. However, the size and composition of the complex vary. Opisthokont MICOS consist of four to six subunits, while in *Trypanosomes* (from the clade discoba (Box 1)), there are nine MICOS components [19]. With the new components came a new function: *Trypanosomes* MICOS include a thioredoxin that is hypothesised to take over the role of the missing oxidoreductase, Mia40, essential for protein import into the intermembrane space in opisthokont mitochondria [20]. In *Toxoplasma*, homologs of MIC60/10 are also identifiable in the genome [21], but no experimental work has addressed MICOS composition and function yet. Like *Trypanosoma*, *Toxoplasma* is also missing a Mia40 homolog [22], thus, it is intriguing to find out if the same convergence occurred in both these organisms, which are models of unrelated clades. Moreover, it would be of interest to discover whether other novel *Trypanosomes* MICOS properties are found in *Toxoplasma* or indeed representatives of other clades, that might point to ophistokont being the outlier as proposed by the *Trypanosomes* MICOS study [20].

## Proteins complexes control inter-organelle interactions via membrane contact sites

While often cell biology textbooks discuss organelles as separate entities, it is known that contact, communication, and exchange between organelles are required for function. The past decade has seen a growing appreciation of the protein complexes mediating these interactions. These so-called membrane contact sites (MCSs) are defined with four general rules: the membranes are in close proximity (but there is no membrane fusion); the juxtaposed membranes are actively tethered; the contact performs a dedicated cellular function, and the MCS contains a MCS-specific proteome/lipidome [23]. Contacts following these rules have been identified between any two organelles studied to date, and the molecular details of numerous MCS have been elucidated. However, as most MCS studies are performed in opisthokonts, the full functional and evolutionary understanding of the role of MCS in eukaryotic biology remains incomplete without the perspective from a broad range of divergent organisms. Pioneering work focused on less well-studied model systems has exposed novel contacts [24] involving a mitochondria-like organelle and ER [25], acidocalcisome and mitochondria [26], and even a contact between the vacuole surrounding intracellular parasites and host-cell organelles [27]. In this part of the review, we will highlight recent advances on the protein complexes mediating MCS between organelles inside *Toxoplasma* (Box 2).

Box 2 **Toxoplasma gondii as a model apicomplexan.**

As the field of eukaryotic cell biology expands the repertoire of model organisms from divergent clades, *Toxoplasma* is established as an effective model for apicomplexan and other groups within the SAR clade, tools and resources that are the best developed than for any other member of the SAR. This includes the below:

• Wide range of genetic manipulation systems [2].
• Relative high efficiency of genetic manipulation due to high transfection rates, a balanced nucleotide composition, and haploidy of relevant life stages.
• Single copy of many organelles including mitochondrion and Golgi, relative large size and typical signature of organelle morphology.
• Relatively ease, low cost, and safe culture.
• Being clinically relevant.

Alt-text: Box 2

## Mitochondrion and ER

ER–mitochondria MCSs are the best-studied contacts, with functions including calcium and lipid exchange and control of mitochondrial dynamics, autophagy, and apoptosis [28]. The first tether reported for this contact is the ER–mitochondria encounter structure (ERMES) discovered in yeast [29]. ERMES was subsequently found in various divergent organisms across the eukaryotic tree while, somewhat surprisingly, not conserved among the opisthokonts [4], highlighting an example of the importance of exploring different systems (Box 1). Another one of those MCS is mediated by the ER–localised inositol trisphosphate receptor (IP3R) and the voltage-dependent anion channel (VDAC), which is an outer mitochondrial membrane porin. In this contact, which was first described in mammalians [30], the proximity between IP3R and VDAC facilitates calcium mobilisation from the ER into the mitochondrion, where calcium moves through a mitochondrial calcium uniporter (MCU) to the lumen. The VDAC-IP3R contact is further mediated by the proteins Grp75 and DJ-1 [31]. The presence of porins eukaryotes raised the question of whether this contact might be broadly conserved [18], however, a study of the role of *Toxoplasma* VDAC points to a divergence [32]. On the one hand, the study identifies structures with the features of ER–mitochondrial MCS in *Toxoplasma* and further shows that these contacts are reduced upon VDAC depletion. On the other hand, however, the depletion and resulting MCS reduction have no effect on calcium homeostasis [32]. Moreover, homologs for IP3R and MCU were lost in apicomplexans [33]. Thus, while a function for VDAC in mediating ER–mitochondrion MCS is supported, a role in calcium homeostasis is not. Furthermore, a partner for this putative tether is yet to be identified. Interestingly, while a VDAC-IP3R MCS is found in *Trypanosomes*, it mediates exchange between the mitochondrion and acidocalcisome rather than the ER [34]. Therefore, it seems that porin mediated mitochondria contacts may assume different roles in divergent organisms.

Another complex proposed to mediate an ER–mitochondrial MCS in yeast is the ER membrane complex (EMC). The ER resident EMC was suggested to interact with the mitochondrial Tom5, and the resulting MCS is thought to facilitate phospholipid transfer between the two organelles in both yeast [35] and mammalians [36]. Like VDAC, most EMC subunits are found in most eukaryotic organisms [37], raising the possibility of a putative common function across eukaryotes. This notion was supported by the report of EMC found in the ER–mitochondrial interface of *Trypanosomes*, where it plays a role in the control of phospholipid synthesis [38].

Of note, EMC, or some of its subunits, are proposed to perform multiple other roles, including acting as an ER transmembrane protein insertase [39]. EMC is composed of up to nine subunits encoded by up to ten genes in different organisms. In yeast, EMC genes could be individually deleted without a major effect on viability, but deletion of two (or more) subunits results in lipid synthesis defect [35]. Interestingly, in *Trypanosomes*, individual gene deletion or depletion affects viability, and some have a drastic effect on lipid synthesis [38]. Single-subunit essentiality seems to be the case also in *Toxoplasma,* where seven of the eight predicted EMC encoding genes are critical for growth, as predicted by a genome wide CRISPR screen (Table 1) and confirmed in our unpublished work (Ovciarikova et al., in prep). Whether this importance for fitness is linked to a role in ER–mitochondria tethering, ER–insertase, or other roles remains to be studied. Complexome profiling in the apicomplexan *P. falciparum* found only seven of the eight predicted EMC subunit homologs in a putative EMC complex and further found the subunit EMC5 also in a separate, unknown, complex [17] supporting the hypothesis of multiple roles.

**Table 1:** . **A summary of the data available for homologs of the EMC components in two model apicomplexans, *Toxoplasma gondii,* and *Plasmodium falciparum***. Fitness scores for *Toxoplasma* are from the study by Sidik et al. [40], and for *Plasmodium falciparum* are from a PiggyBac screen [41]. In both, lower score reflects higher contribution to fitness. Localisation prediction is based on localisation of organelle proteins by isotope tagging in *Toxoplasma* [42]. Presence in a complexome analysis provides evidence for belonging to the same complex [17].

	Toxoplasma gondii			Plasmodium falciparum		
	Gene ID	Fitness-conferring score	Localisation prediction	Gene ID	Present in complexome analysis	Fitness-conferring score
EMC1	TGME49_205740	−5.03	ER	PF3D7_0811200	Yes	−3.23
EMC2	TGME49_267840	−4.08	ER	PF3D7_1410000	Yes	−3.42
EMC3	TGME49_230100	−3.02	ER	PF3D7_1360200	Yes	−2.81
EMC4	TGME49_259000	−4.77	ER	PF3D7_1435400	Yes	−3.3
EMC5	TGME49_293200	−2.68	ER	PF3D7_0306700	Yes	−3.39
EMC6	TGME49_239690	−3.57	No data	PF3D7_1214400	No	−2.76
EMC7	TGME49_243390	−1.71	ER	PF3D7_1310500	Yes	−3.08
EMC8	TGME49_249310	−4.1	ER	PF3D7_1139900	Yes	−2.84

## Nuclear-mitochondrial contacts

**Figure 1:**
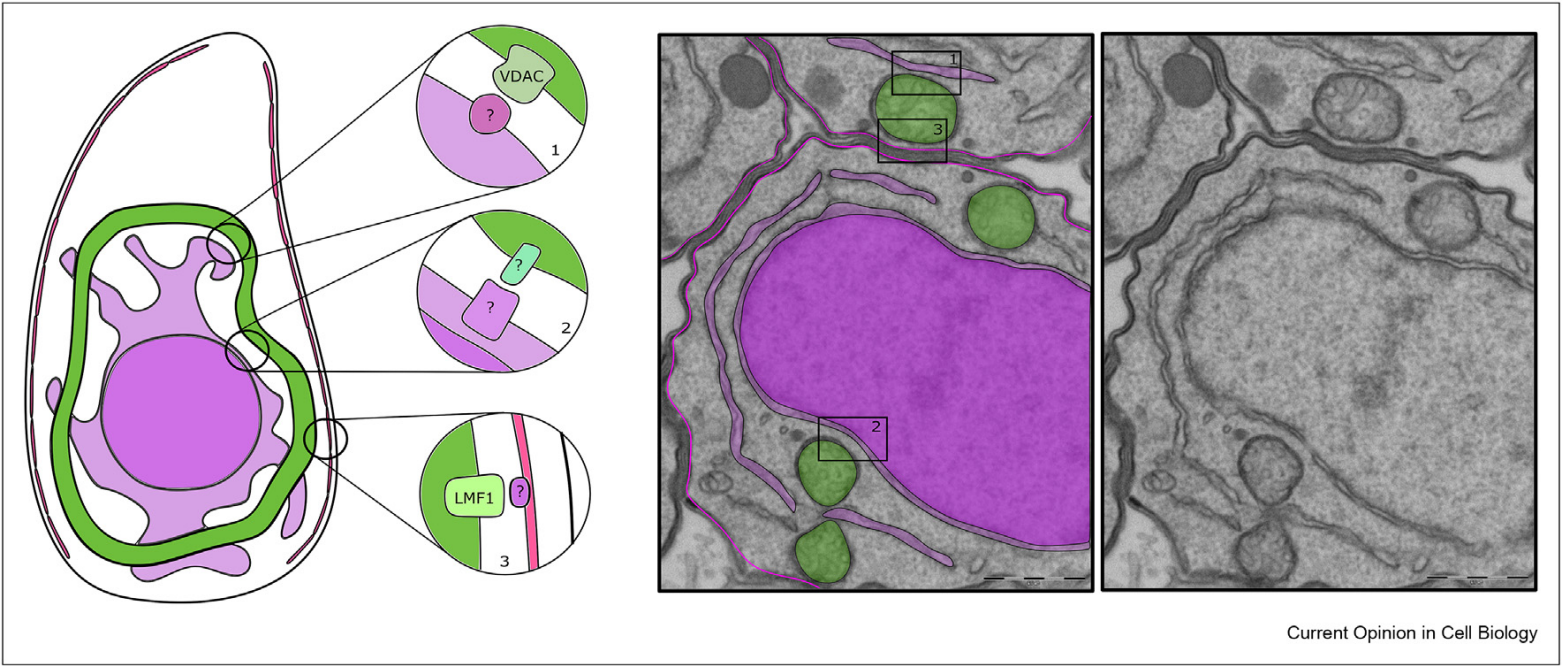
**. Putative membrane contact sites observed and described in *Toxoplasma gondii*.** A scheme of a single *Toxoplasma* tachyzoite (left) showing mitochondrion (green), ER (light purple), nucleus (dark purple), and inner membrane complex (IMC) (pink). Contact sites discussed in the main text are highlighted in circles with identity of proposed components shown: (1) ER–mitochondrion; (2) nucleus–mitochondrion; and (3) mitochondrion–IMC. EM images of *Toxoplasma* (middle/right) showing examples of organelle proximity that provide evidence for the contacts shown in (A), whereby A and B are colour coordinated, and C has no added colours for clarity.

Recent studies have started addressing the composition and function of nuclear–mitochondrial MCSs, where the first characterised contact might be involved in phospholipid homeostasis [43]. Putative mitochondrial–nucleus contacts were observed in the apicomplexans *Toxoplasma* (Figure 1, and our unpublished work) and *Plasmodium* [44]*.* In the latter, nuclear–mitochondrial proximity is enhanced in parasites that survived treatment with artemisinin, a potent anti-malarial drug, and it was proposed that the new contacts may support mitochondrial retrograde signalling, resulting in transcriptional changes in the nucleus as a survival mechanism [44]. Likewise, in cancer cells, these newly described contacts were proposed to promote survival via a mitochondrial retrograde response [45]. Identifying the tethers mediating these contacts in a broad array of divergent organisms is the next step for the field.

## Contacts described only in *Toxoplasma*

Apicomplexans and other alveolates contain a network of membrane sacs likely of ER–Golgi origin, named the inner membrane complex (IMC) in apicomplexans. In *Toxoplasma*, MCSs are seen between the IMC and the mitochondrion [46]. Microscopy observations showed that mitochondrion–IMC contacts change dynamically during the *Toxoplasma* lytic cycle, which coincides with mitochondrial morphological changes from its typical lasso shape when the parasites are inside their host into a ball-shape when they are out [46]. A parasite specific protein of the mitochondrial outer membrane, named lasso maintenance factor 1 (LMF1), acts as a bridge between the two organelles, whereby LMF1 depletion results in loss of the lasso shape [47]. This mitochondrion positioning is important for cell division and for parasite proliferation *in vitro* [47]. Whether LMF1 acts as a direct tether and what might be its partner/s in the IMC remains to be discovered.

Another organelle specific to Apicomplexa and related groups is their secondary plastid, the apicoplast. Proximity between mitochondrion and apicoplast [48] and between the apicoplast and the ER [49] have been detected by electron microscopy, which might indicate contacts. An apicoplast–ER contact was also reported in a study of an apicoplast-localised two-pore channel (TgTPC) [50]. TgTPC depletion reduces these contacts and affects calcium homeostasis, thus this putative contact is proposed to allow calcium exchange between these two organelles [50]. Whether TgTPC is a tether component and what might be the ER partner remains to be explored.

## Conclusions

Like many fundamental cell biology processes, the role of membrane complexes in controlling organelle function is best studied in common model organisms, largely of the opisthokont clade. Often, complex conservation between yeast and mammals is termed ‘evolutionarily conserved’ and even seen as ‘gold standard’ for a given complex's composition or function. However, studies in divergent organisms reveal divergent traits and define true evolutionary conservation, thus highlighting the importance of stirring away from this simplified view. In this context, research in the divergent *Trypanosomes* has provided numerous examples for unexpected divergence in biology. While studies of organisms from other divergent clades are lagging, *Toxoplasma* is established as an effective and relevant model organism for the SAR clade or for groups within it for addressing questions of divergent cell biology (Box 2).

As the picture widens, we find that single cell parasites do not always have ‘less’ subunits of functional complexes compared to their opisthokont parallel. Rather the parasite complexes may consist of other unrelated proteins, as is likely the case for the VDAC mediated mitochondria–ER MCS in *Toxoplasma*. Or, the divergent complexes have more subunits, as seen in the respiratory chain enzymes of apicomplexans. This comparison provides powerful insights. For example, the higher number of components of apicomplexan ATP synthase might be linked to the reduction of its mitochondrial genome. In support of this notion, the *Toxoplasma* ATP synthase structure exposed reduced hydrophobicity of a truncate and nuclear encoded subunit a, enabling its post translational translocation into the mitochondrion and compensated for by interaction generated with novel subunits.

Thus, eukaryotic biology should not be defined in comparison to what has been found first in opisthokont, as often is the case, but rather revised in a broad context, when each complex is studied in a divergent repertoire of systems. This would enhance the ability of the cell biology field to understand the fundamental role of biological processes and their evolution.

## Conflicts of interest

Nothing declared.
